# All of this and he can cook, too…

**DOI:** 10.1007/s40037-012-0031-2

**Published:** 2012-11-09

**Authors:** Jan van Dalen

**Affiliations:** Coordinator of Communication Skills Training and Assessment, Skills Laboratory, Maastricht University, Maastricht, the Netherlands

**Keywords:** Karolinska Award, Research

## Abstract

Professor Cees van der Vleuten has been awarded the 2012 Karolinska Insitutet Prize for Research in Medical Education for his research in evaluation and assessment of medical competences.

 Interview with Professor Cees van der Vleuten, Maastricht University

Cees van der Vleuten (1956) accepted a position as a psychometrician at the Department of Educational Development and Research at Maastricht University in 1982. In 1992 he was appointed as Professor of Education and accepted the position of Chair of the Department. Since 2005, he is Scientific Director of the School of Health Professions Education (SHE, www.maastrichtuniversity.nl/she).

Professor van der Vleuten’s area of expertise lies in evaluation and assessment. He has published widely on these topics, holds numerous academic awards for his work, including several career awards. He is an international consultant at about 70 institutions and has supervised >50 doctoral graduate students. A full curriculum vitae can be found at http://www.fdg.unimaas.nl/educ/cees/CV/.

The Karolinska Instuitutet Prize for Research in Medical Education is awarded for outstanding research in medical education. The purpose of the prize is to recognize and stimulate high-quality research in the field and to promote long-term improvements of educational practices in medical training.

At the occasion of his acceptance of this award, Professor van der Vleuten has found time to answer some of *Perspectives on Medical Education*’s questions.

*Professor van der Vleuten, many congratulations on the Karolinska Award. How did you get involved in medical education?*

That was sheer coincidence. It had to do with a decline, at that time, in academic positions in psychology, in which I graduated, and my own ambition to pursue a career in academia. A position became available at the new medical school in Maastricht, for which I applied. When the committee started to question me about IRT models, I advised them to hire someone else, but instead they hired me.

The great thing about Maastricht University is its strong interdisciplinary character. The institution’s culture encouraged collaboration across disciplines and professions. At that time Maastricht University was a pioneer school, characterized by a dedicated spirit, and we were a growing institution. After a year of computer programming I started to advise colleagues on their research designs. This further developed into my interest in assessment. Following in the footsteps of Henk Schmidt, my predecessor at our Department, I collaborated with Scheltus van Luijk in research on the Objective Structured Clinical Examination (OSCE) at Maastricht.

The annual Summer Course of our school attracted many visitors who were involved in curriculum innovation. I have always enjoyed meeting people with their own experiences and questions, so I gladly participate in the Summer Courses. As a consequence, every Summer Course resulted in a number of invitations to visit and provide consultation overseas.

Starting your career at Maastricht includes the risk of taking our benefits for granted. Learning about the very different circumstances abroad taught me to remain modest and keep my feet on the ground. Looking around in other institutions is still a very important aspect of my consultations.

*What are you most proud of in your career?*

I have contributed to developing a community of practice in health professions education. This health education community is now a large group of professionals exchanging educational practices and research findings, both nationally and internationally. We are an open and welcoming community, by means of conferences, journals and staff and student exchanges. Conferences and journals have their profiles, but together they cover all relevant areas. They provide a platform in which members of this community can consult each other.

It is important that both theory and practice are represented in these platforms; in other fields we witness that there is a huge risk of a gap between research and practice. Our platforms serve to avoid that risk. However, we must be careful that we do not overestimate research at the expense of learning from experiences in practice. We must be aware that it is easy to attach more weight to research and to neglect practical experience. The title of my inaugural address was ‘Beyond Intuition’: that is what I am after. We should constantly ask ourselves why we do what we do, and find arguments for the optimal choice.

*Which recommendations do you have for educational renewal?*

To move ‘beyond intuition’! All too often we teach in a way that is only based on tradition; because we have always done it that way. We must appreciate that providing information is not sufficient to establish learning. We know for sure that three aspects are needed for real learning: elaboration, coaching, and, consequently, observation and feedback. This requires dedication and active involvement of teachers, it does not come by itself. But then again, some aspects of the medical curriculum can be organized more efficiently. Isn’t it ridiculous that all the thousands of medical schools around the world individually teach the same basic and clinical sciences? Wouldn’t it be much more efficient if teachers of these topics were to exchange their lessons and teaching aids? Exchange is so easy nowadays, with all our electronic facilities.

Assessment is not separate from teaching, it is a tool for teaching. If it is, we see a lot of negative affects. The old dictum ‘assessment drives learning’ should then be: assessment drives non-learning! Good assessment *helps* learning, by focusing on what we want our students to learn.

*And which recommendations could you give for educational research?*

Why did you separate these two questions? They are two sides of the same coin. Both practice and research should support good learning environments. Research should influence the learning environment and the teaching and learning practice fertilizes good research. Good research is done by people who know the teaching and learning practice.

I am worried about the strong one-sided emphasis on research; theory and practice should reinforce and reciprocate each other. One cannot be without the other. Curriculum innovation should not become a ritual, it must remain a rational process.

*And finally, how do you manage your responsibilities in life? Where do you find the time?*

Eh, OK, I work a lot, but I am also the cook in our household, and we have four children, and I like to sail…

I think it can be compared to top sport. I succeed in protecting the time that is for my family. Nobody interferes with our holidays. I protect the time that I allocate for writing. But most of all, I have very good people around me. My staff take care of many things that I then don’t have to worry about…

*Would you recommend such a professional life to your children?*

I asked them that very question, and they responded by challenging me about my working hours. They stated that they will never opt for such a working life themselves. But you know what’s interesting: I see them going the same way…

